# 535 Pre-operative Expectations, Post-operative Satisfaction and Patient Directed Priorities for Clinical Burn Research

**DOI:** 10.1093/jbcr/irac012.164

**Published:** 2022-03-23

**Authors:** Shyla K Bharadia, Jenny Horch, Lindsay Burnett, Zheng Yu, Hua Shen, Vincent Gabriel

**Affiliations:** University of Calgary, Calgary, Alberta; Foothills Hospital, Calgary, Alberta; Alberta Health Services, Calgary, Alberta; University of Calgary, Calgary, Alberta; University of Calgary, Calgary, Alberta; University of Calgary, Calgary, Alberta

## Abstract

**Introduction:**

Patients receiving split thickness skin grafting due to deep burns are left with scarring and chronically dysfunctional skin at the graft site. Given evidence that patients’ pre-operative expectations mediate post-operative outcomes and satisfaction, we sought to describe burn patients’ experience, expectations, and satisfaction with their skin graft, and their views towards a future cell-based clinical trial to improve their graft, over time. We also aimed to identify graft outcome measures for use in future studies.

**Methods:**

This study was approved by our university's research ethics board. All participants provided written and informed consent. Data were collected via patient questionnaires pre-operatively, one, and three months post-operatively.

**Results:**

Most patients had small burns. Expectations of graft function were consistent pre- and post-operatively. Expectations of graft appearance showed significant decrease over time (β _1_ = -0.290, p = 0.008). Significant improvements in skin function (β _1_ = 0.579, p = 0.000) and appearance (β _1_ = 0.247, p = 0.025) at the wound site during recovery were observed, although patients noted great difference between grafted and normal skin. Patient satisfaction with their graft did not change significantly over time. Patients were willing to participate in a cell-based clinical trial that may improve graft symptomology. They prioritized diminished scarring, redness, and improved sensation and elasticity as the most salient aspects of grafts to be enhanced by cell-based therapy.

**Conclusions:**

Patient graft concerns changed over time; outcome measures in trials advancing skin grafting should reflect chronic, patient prioritized limitations.

